# Sexual fantasies, subjective satisfaction and quality of sexual life in patients of sexual dysfunction: A comparative study

**DOI:** 10.1192/j.eurpsy.2021.450

**Published:** 2021-08-13

**Authors:** N. Ohri, A. Dubey, G. Vankar, P. Rathi, A. Gill

**Affiliations:** 1 Psychiatry, New Life Hospital, Varanasi, India; 2 Psychiatry, Sri Aurobindo Medical College and PGI, Indore, India; 3 Psychiatry, Parul Institute of Medical Scieces, Vadodara, India

**Keywords:** Sexual Dysfunction, sexual fantasy, Sexual Quality of Life, Sexual experience

## Abstract

**Introduction:**

Exploring the ways in which sexual fantasies may affect sexual experience and satisfaction is of relavence in the clinical setting involving sexual dysfuntion.

**Objectives:**

To observe how the sexual fantasy scores differ in their relationship with sexual satisfaction, experience and quality between sexual dysfunction cases and normal controls.

**Methods:**

Scales included: Wilson’s sex fantasy questionnaire (WSFQ), Arizona Sexual Experience Scale (ASEX), Sexual Quality of life Questionnaire (SQoL), and a subjective sexual satisfaction meter. Differences in resposes of both groups on WSFQ (item-wise and domain-wise) were analysed using T-tests. Two-way ANOVA was applied to see how other scales affected sexual fantasy.

**Results:**

Cases scored significantly higher on ASEX scale, and low on satisfaction, SQoL and WSFQ

**Table:** 

	Cases N=1OO	Controls N=100	t-test
Satisfaction Mean(sd)	4.27(1.85)	7.82(1.31)	t=3.052;df=198,p=0.0026
Asex	17.52(4.73)	8.28(1.34)	t=15.24;df=198,p<0.0001
SQoL	29.41(12.12)	49.5(6.67)	t=14.52;df=198,p<0.0001
WSFQ	26.80(17.61)	30.59(15.32)	t=1.62,df=98,p=0.106

Majority of WSFQ responses, both in cases and controls, fell in the intimate and impersonal domains. Sexual fantasy scores and sexual satisfaction had a strong positive and significant correlation in controls but no linear correlation in the case-subjects. sexual fantasy scores contributed to 5.7% of difference in the scores of SQoL between groups. Major variance in scores of satisfaction in our subjects depended on presence or absence of sexual dysfunction(46.5%)but sexual fantasies also contributed to 8.8% of the variance.

**Conclusions:**

The study showed that fantasies contribute to positive sexual outcomes only in absence of sexual dysfunction. ANOVAanalysis revealed that in case-subjects sexual satisfaction briefly increases initially with increase in fantasy scores but starts to decline as fantasies increase.

**Disclosure:**

No significant relationships.

